# Supplementary material for: Multi‐dataset identification of innovative feature genes and molecular mechanisms in keratoconus
Source: J Cell Mol Med. 2024 Sep 19;28(18):e70079. doi: 10.1111/jcmm.70079 (PMC11412914; doi:10.1111/jcmm.70079)
Supplement: Supplementary file 2 — Material S2. [file JCMM-28-e70079-s002.zip › 7.GSEA analysis/6.SNORA74B/KEGG_GLYCOSAMINOGLYCAN_DEGRADATION.html]

Details for gene set KEGG\_GLYCOSAMINOGLYCAN\_DEGRADATION[GSEA]

|  || Dataset | SNORA74B.group.cls#h\_versus\_l |
| Phenotype | group.cls#h\_versus\_l |
| Upregulated in class | h |
| GeneSet | KEGG\_GLYCOSAMINOGLYCAN\_DEGRADATION |
| Enrichment Score (ES) | 0.24921085 |
| Normalized Enrichment Score (NES) | 0.7857457 |
| Nominal p-value | 0.73006135 |
| FDR q-value | 1.0 |
| FWER p-Value | 1.0 |
Table: GSEA Results Summary

  

Fig 1: Enrichment plot: KEGG\_GLYCOSAMINOGLYCAN\_DEGRADATION      
 Profile of the Running ES Score & Positions of GeneSet Members on the Rank Ordered List

  

| SYMBOL | TITLE | RANK IN GENE LIST | RANK METRIC SCORE | RUNNING ES | CORE ENRICHMENT || 1 | HS3ST3A1 | na | 1272 | 0.442 | 0.2492 | Yes |
| 2 | HYAL3 | na | 10546 | 0.082 | 0.0354 | No |
| 3 | ARSB | na | 11971 | 0.060 | 0.0334 | No |
| 4 | HS3ST3B1 | na | 12568 | 0.052 | 0.0500 | No |
| 5 | HYAL4 | na | 13407 | 0.043 | 0.0534 | No |
| 6 | HGSNAT | na | 14220 | 0.033 | 0.0516 | No |
| 7 | IDUA | na | 14850 | 0.026 | 0.0503 | No |
| 8 | SGSH | na | 15117 | 0.023 | 0.0577 | No |
| 9 | HYAL1 | na | 15967 | 0.015 | 0.0429 | No |
| 10 | NAGLU | na | 17211 | 0.003 | 0.0088 | No |
| 11 | GALNS | na | 17227 | 0.002 | 0.0099 | No |
| 12 | HYAL2 | na | 17676 | -0.002 | -0.0015 | No |
| 13 | HEXA | na | 17794 | -0.004 | -0.0026 | No |
| 14 | GNS | na | 18066 | -0.007 | -0.0060 | No |
| 15 | GUSB | na | 19513 | -0.023 | -0.0327 | No |
| 16 | GLB1 | na | 22952 | -0.065 | -0.0901 | No |
| 17 | HEXB | na | 23118 | -0.067 | -0.0514 | No |
| 18 | IDS | na | 25354 | -0.104 | -0.0482 | No |
| 19 | HPSE | na | 26693 | -0.132 | -0.0012 | No |
| 20 | HPSE2 | na | 27653 | -0.155 | 0.0716 | No |
| 21 | SPAM1 | na | 29341 | -0.204 | 0.1552 | No |
Table: GSEA details [plain text format]

  

Fig 2: KEGG\_GLYCOSAMINOGLYCAN\_DEGRADATION      
 Blue-Pink O' Gram in the Space of the Analyzed GeneSet

  

Fig 3: KEGG\_GLYCOSAMINOGLYCAN\_DEGRADATION: Random ES distribution      
 Gene set null distribution of ES for **KEGG\_GLYCOSAMINOGLYCAN\_DEGRADATION**
